# Solvent Annealing Influence of PEDOT on Its Electrochemical and Electrochromic Properties

**DOI:** 10.3390/nano15211620

**Published:** 2025-10-24

**Authors:** Kaiwen Lin, Yuying Jiang, Qinran Chen, Wangdaiqi Kong, Ruiyu Luo, Qianhui Zhou, Hao Liu

**Affiliations:** 1 Department of Materials and Food, University of Electronic Science and Technology of China Zhongshan Institute, Zhongshan 528402, China; 2 Hefei Institute for Public Safety Research, Tsinghua University, Hefei 230601, China

**Keywords:** PEDOT, solvent annealing, electrochemistry, electrochromic

## Abstract

The study of effect of solvent annealing on the optoelectronic properties of polymers is not new research hotspot, but the influence of solvent annealing on the electrochemical and electrochromic properties of PEDOT remains unexplored. This paper investigates the effects of three different solvents—chlorobenzene (CB), tetrahydrofuran (THF), and dimethylformamide (DMF)—on the self-assembly of PEDOT films and compares their thermal, morphological, electrochemical, and electrochromic properties. PEDOT annealed with DMF exhibits a highly crystalline film morphology, which increases the difficulty of ionic doping/undoping and leads to suboptimal electrochemical and electrochromic stability. After CB annealing, PEDOT forms a relatively gentle melting peak. In addition to a certain degree of crystallinity, the polymer film also exhibits cracking, which severely impairs the electrochromic performance. After THF annealing, PEDOT exhibits a gentler melting peak, a surface morphology that is more favorable for electrochemical and electrochromic performance, ultimately achieving an optical contrast of 28%, the fastest response time of 1.1 s, and the highest coloration efficiency of 184 cm^2^ C^−1^. The impact of solvent annealing on PEDOT’s electrochromism is significantly different, which will guide the electrochemical and electrochromic properties of PEDOT analogs and derivatives under the influence of different solvents.

## 1. Introduction

Conjugated polymers have shown great application potential in flexible electronics, energy storage, sensor, and intelligent display fields due to their unique combination of conductivity and processability [1,2,3,4,5]. Among them, poly(3,4-ethylenedioxythiophene) and its derivatives (PEDOTs) have become a core material for electrochemical and electrochromic devices, thanks to its excellent chemical stability, high conductivity, and reversible redox properties [6,7,8].

PEDOT was synthesized by Bayer AG in 1988, which is functionalized with ethylenedioxy at the 3- and 4-positions of the thiophene ring, which has drawn most of the attention in both academic studies and industrial applications [9]. The microstructure of materials is crucial to determining their macroscopic properties. The microstructural characteristics of PEDOTs, such as segment arrangement, crystallinity, and morphology, directly affect charge transport efficiency, ion diffusion rate, and optical modulation ability [10,11]. As a simple and efficient self-assembly regulation method, solvent annealing can achieve precise control of material microstructure by inducing the rearrangement of polymer segments in a solvent atmosphere [12,13,14,15]. Solvent annealing is widely applied in organic optoelectronic materials and devices, mainly including organic photovoltaics, organic field-effect transistors, light-emitting diodes, and electrochromics [16,17,18,19,20]. Different solvents vary significantly in polarity, solubility parameters, and evaporation rate, which can alter the intermolecular interactions and assembly kinetics of PEDOTs, thereby forming diverse microstructures (such as nanofibers, particles, or lamellar stacks), and ultimately leading to obvious differences in its electrochemical activity (e.g., redox peak position, specific capacitance) and electrochromic properties (e.g., response time, contrast ratio, cycling stability) [19,21,22,23].

At present, most studies on PEDOTs properties focus on doping modification or composite system construction, while systematic research on the structure–activity relationship between solvent annealing-induced self-assembly behavior and electrochemical/electrochromic properties remains to be further explored [7,19,21,24]. Clarifying the regulation mechanism of different solvents on PEDOTs self-assembled structures and revealing the intrinsic correlation between microstructural evolution and performance differences are of great theoretical value and practical significance for guiding the preparation of high-performance PEDOT-based devices.

In this paper, PEDOT is taken as the research object, and solvents with different boiling points and polarities parameters are used for solvent annealing treatment to systematically study the changes in the micro-morphology of PEDOT after annealing. Then through electrochemical tests such as cyclic voltammetry, as well as ultraviolet–visible spectroscopy analysis, the influence of solvent types on its electrochemical activity and electrochromic properties is investigated. Finally, the structure–activity relationship between solvent annealing, structure, and performance is established, providing experimental basis and theoretical guidance for optimizing the performance of PEDOT-based electrochemical and electrochromic devices. As far as we know, this would be the first study of PEDOT-based solvent annealing influence on its electrochemical and electrochromic properties.

## 2. Experimental Section

### 2.1. Materials

3,4-Ethylenedioxythiophene (EDOT), tetrabutylammonium hexafluorophosphate (TBAPF_6_), chlorobenzene (CB, boiling point: 132 °C, dielectric constant: 5.7, polarity: 2.7 D), tetrahydrofuran (THF, boiling point: 66 °C, dielectric constant: 7.5–7.6, polarity: 4.2 D), dimethylformamide (DMF, boiling point: 153 °C, dielectric constant: 36.7–37.4, polarity: 6.4 D), and acetonitrile (CH_3_CN) were purchased from Shanghai Energy & Chemical Company (Shanghai, China). All these chemicals are used directly without further purification.

### 2.2. Characterization

Electrochemical experiments, spectroelectrochemistry, and kinetic studies were recorded on a spectroelectrochemical workstation of Xi Pu Guang Dian (XP-SEC-BAC, Guangzhou, China). The surface morphology of polymer films was observed by using a super depth-of-field 3D video microscope DVM6 of Leica (Wetzlar, Germany). Thermogravimetric analysis (TGA) and differential scanning calorimetry (DSC) were performed using Ta Instruments (New Castle, DE, USA) with a scan rate of 20 °C min^−1^ under a nitrogen flow.

### 2.3. Electrochemical Tests and Polymerization

All the electrochemical experiments were performed in a one-compartment cell with the use of spectroelectrochemical workstation of Xi Pu Guang Dian (XP-SEC-BAC, Guangzhou, China). An Ag/AgCl electrode was used as the reference electrode, a platinum wire as the counter electrode, and an indium tin oxide (ITO) as the working electrode. TBAPF_6_ dissolved in organic solvents (0.1 M) was employed as the supporting electrolyte. Electropolymerization of the EDOT (0.01 M solution) was performed at a scan rate of 100 mV s^−1^ for 10 cycles under ambient conditions. The obtained PEDOT (0.8 × 2.5 cm^2^), both alone and with CB, THF, and DMF solutions, was placed in a sealed container, but it should not be in contact, for 4 h. Cyclic voltammograms of PEDOT films were obtained using the polymer electrodes setup in the monomer-free CH_3_CN solution containing 0.1 M TBAPF_6_. For spectroelectrochemical and electrochromic experiments, the polymer films were electrodeposited in the same fashion on an ITO-coated glass electrode and their UV-Vis spectra at different applied potentials were recorded.

Optical absorption measurements provide energy difference values between the previously described band edges. The *E*_g,opt_ of all polymers was determined from their onset absorption (*λ*_onset_) from UV-vis spectra with the following equation:

$$E_{g,opt}=1240\;/\;\lambda_{onset}\left(\mathrm{eV}\right)$$

### 2.4. Spectroelectrochemical and Electrochromic Studies

Spectroelectrochemistry and kinetic studies of PEDOT were recorded on a spectroelectrochemical workstation of Xi Pu Guang Dian (XP-SEC-BAC, Guangzhou, China). An Ag/AgCl electrode as the reference electrode, indium tin oxide (ITO)-coated glass slide as the working electrode, and a Pt wire as the counter electrode in a transparent cuvette. These characterizations were all performed in TBAPF_6_-CH_3_CN (0.1 M) electrolytes.

The potentials were alternated between the reduced and oxidized states with a residence time of 10 s. The optical contrast at the specific wavelength (*λ*) was determined by ∆*T* values of polymer films, using the following equation:

$$∆T=|T_{ox}-T_{red}|$$

The response time (*t*) refers to the time it takes for the transmittance to change by 95% during the transition between the oxidized and reduced states of the electrochromic material. The response time is often used to characterize the color-changing speed of electrochromic materials. The shorter the response time, the faster the electrochromic speed.

The coloration efficiency (*CE*) is defined as the relation between the injected/ejected charge as a function of electrode area (*Q_d_*) and the change in optical density (∆*OD*) at the specific wavelength (*λ*) of the sample as illustrated by the following equation:

$$CE=\;∆OD\;/\;Q_{d}$$

$$∆OD=log(T_{ox}/T_{red})$$

## 3. Results and Discussion

### 3.1. The Electrochemical Properties of EDOT

**Figure 1:**
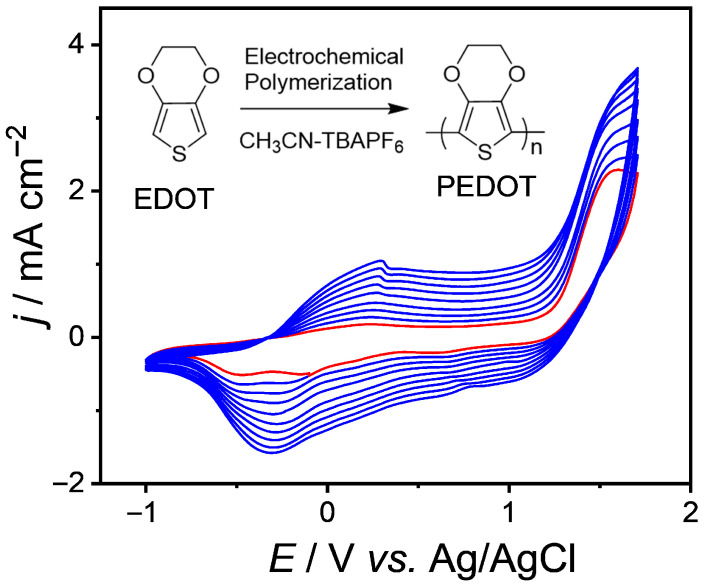
Cyclic voltammograms of EDOT (0.01 mol·L^−1^) in TBAPF_6_-CH_3_CN solution (0.1 M) with scan rate: 100 mV·s^−1^.

The cyclic voltammetry is often used to reveal changes in behavior of polymers in the field of electrochemistry. By analyzing the first cyclic voltammetric curve, it is found that the redox process of EDOT exhibits more pronounced oxidation and reduction peaks, with the oxidation peak potential at 0.2 V and the reduction potential at −0.25 V (Figure 1). The coupling reactions (C-C coupling) rapidly occur between the highly reactive cationic radicals generated by oxidation, forming dimers and deprotonating [25]. The first cycle oxidation-reduction peak current density of EDOT monomer cyclic voltammetry (red line) is low. As the number of scanning cycles increases, the peak current density shows an increase (blue lines). This reflects the growth changes in the PEDOT on the electrode surface. With an increasing number of scanning cycles, the monomer continuously undergoes electrochemical polymerization reactions on the electrode surface, causing the quality and thickness of the PEDOT to gradually increase. Additionally, with the increase in the number of cyclic scans, a phenomenon is observed where the oxidation peak potential shifts to a more positive potential. This is attributed to the formation of dimers or oligomers during the initial stages of polymerization, increasing resistance or hindering ion diffusion caused by the thicker polymer film [26].

### 3.2. Thermal Analysis

**Figure 2:**
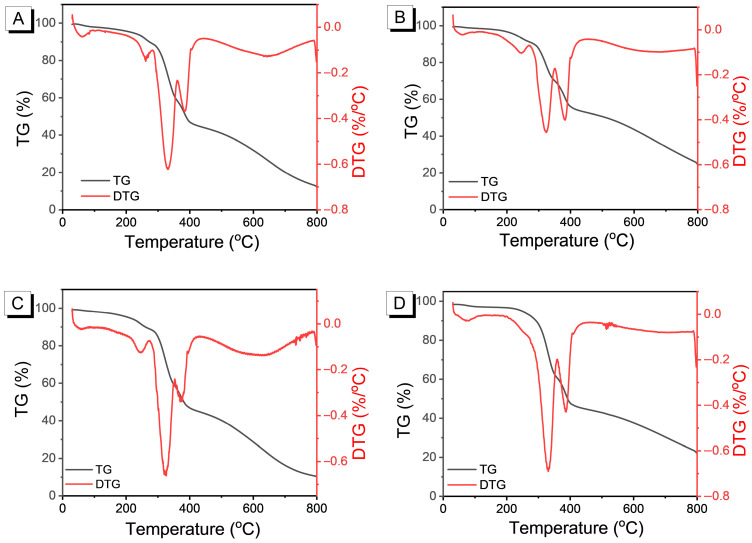
TG and DTG of PEDOT films at room temperature without post-treatment (**A**) and treated with CB (**B**), THF (**C**), and DMF (**D**).

Thermal stability determines the temperature range in which a polymer can be used under different conditions. Since the thermal decomposition behavior of PEDOT in solvent annealing under different polar conditions directly affects its applicable temperature range, it was subjected to thermogravimetric analysis, which was used to characterize the thermal properties of PEDOT in solvent annealing under different polar conditions, and the results are shown in Figure 2.

The test data show that the decomposition temperatures of PEDOT films at 5% mass loss at room temperature are 216 °C without post-treatment, 227 °C after chlorobenzene (CB) treatment, 208 °C after tetrahydrofuran (THF) treatment, and 247 °C after N,N-dimethylformamide (DMF) treatment, respectively. The PEDOT treated with CB exhibited the excellent thermal stability because CB is a very effective π-π stacking-promoting solvent. The electrical conductivity and stability of PEDOT are highly dependent on the strong π-π stacking structure formed between its conjugated main chains. The boiling point of THF is low, and the volatilization rate is too fast. During the annealing process, THF evaporates too quickly, and the PEDOT molecular chains are “frozen” before they can undergo sufficient rearrangement and crystallization. This results in low crystallinity, disordered structure, and a higher number of defects in the film, leading to decreased thermal stability [27]. DMF molecular structure contains carbonyl and methyl groups, and dipole interactions with EDOT units in PEDOT, resulting in stable structure. Meanwhile, DMF has a boiling point of 153 °C, which is much higher than that of CB and THF. The evaporation rate of DMF is positively correlated with the rate of segmental rearrangement. As the solvent “gradually departs,” PEDOT has ample time to complete π-π stacking and lamellar growth, resulting in high crystallinity and the best thermal stability.

**Figure 3:**
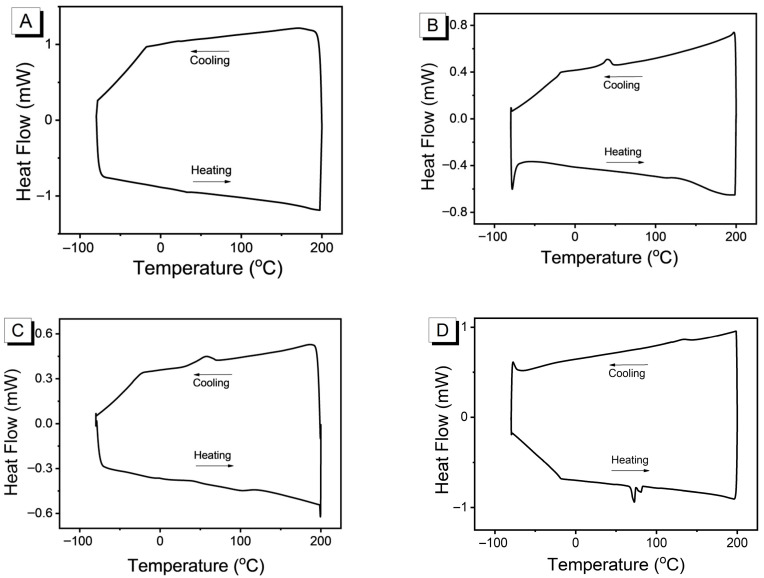
DSC of PEDOT films at room temperature without post-treatment (**A**) and treated with CB (**B**), THF (**C**), and DMF (**D**).

To objectively evaluate the thermal properties of the PEDOT, differential scanning calorimetry (DSC) was employed to measure the power difference (i.e., the heat flow rate difference) between the PEDOT and the reference as a function of temperature or time, thereby providing insight into the energy transfer that occurs during physical or chemical changes. The DSC curves of PEDOT were shown in Figure 3.

The DSC curve of PEDOT films at room temperature without post-treatment is extremely flat with no discernible thermal transition features (Figure 3A). PEDOT primarily exists in an amorphous state, with molecular chains randomly entangled. Following CB annealing, in the heating process, the curve tends to flatten without a distinct melting peak, while in the cooling process, a relatively pronounced but not sharp crystallization peak appears (Figure 3B). This annealing provides PEDOT molecular chains with sufficient mobility and time to undergo local rearrangement, forming more ordered regions (enhancing intermolecular interactions such as π-π stacking). This partially ordered structure enables chain segments to initiate motion at lower temperatures, yet the degree of order remains insufficient for complete crystallization, thus avoiding sharp melting peaks. This moderate level of order proves highly advantageous. It simultaneously provides the chain segment mobility required for ionic transport and the intermolecular pathways necessary for electronic transport (π-π stacking) [28]. Following THF annealing, a crystallization peak similar but broader than that after CB solvent annealing was obtained (Figure 3C). Its interaction with PEDOT may be sufficiently strong to induce the formation of a highly ordered crystalline structure during annealing. DMF possesses strong solvent capabilities and interaction with PEDOT chains. Annealing in DMF induces a higher degree of crystallinity or forms crystals with different polymorphs. This results in a sharper, more pronounced melting peak (Figure 3D). Although extremely high crystallinity has high conductivity, it may impair the ion transport rate [29].

### 3.3. Morphology

**Figure 4:**
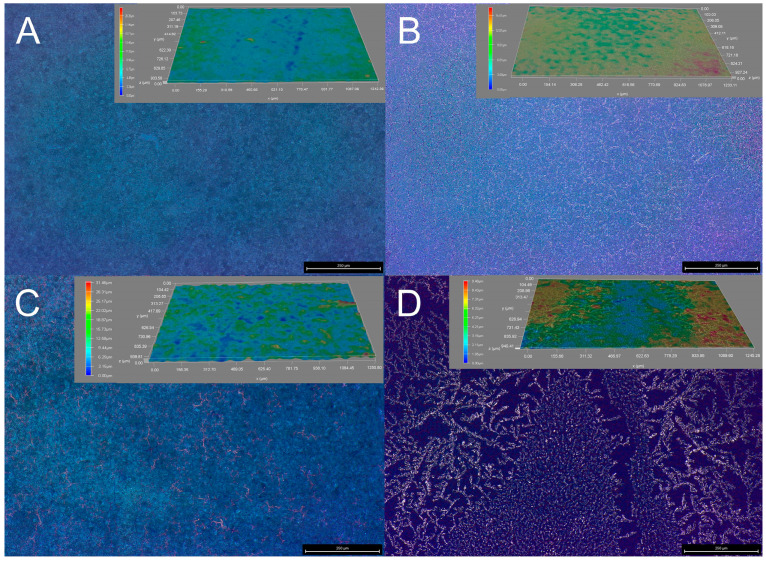
Three-dimensional video microscope images of PEDOT without post-treatment (**A**), and treated with CB (**B**), THF (**C**), and DMF (**D**), respectively. The illustrations are height maps of the respective PEDOT films.

The morphology of the polymer has a certain effect on its conductivity, redox activity, electrochromic properties, etc. The surface morphology can be observed by scanning with a 3D video microscope DVM6 of Leica, and the illustrations are height maps of the respective polymer films, which was shown in Figure 4. PEDOT without post-treatment exhibits a relatively smooth and soft surface morphology (Figure 4A). After solvent annealing, all PEDOT films show some degree of crystallization; in particular, the film annealed in DMF precipitates distinct colorless crystals (Figure 4D), consistent with the DSC results. However, such a surface morphology may disrupt the transport of ions, thereby compromising the electrochemical redox activity, stability, and electrochromic kinetic stability of the PEDOT film. It is worth noting that after CB annealing the PEDOT film develops noticeable cracks, which may impair subsequent electrochromic performance (Figure 4B). In terms of thickness, PEDOT without post-treatment and that treated with THF possess thicker films and similar colors, whereas the DMF-annealed film is darker and exhibits a more pronounced color change during redox switching, leading to higher optical contrast.

### 3.4. Redox Activity and Stability of PEDOT

**Figure 5:**
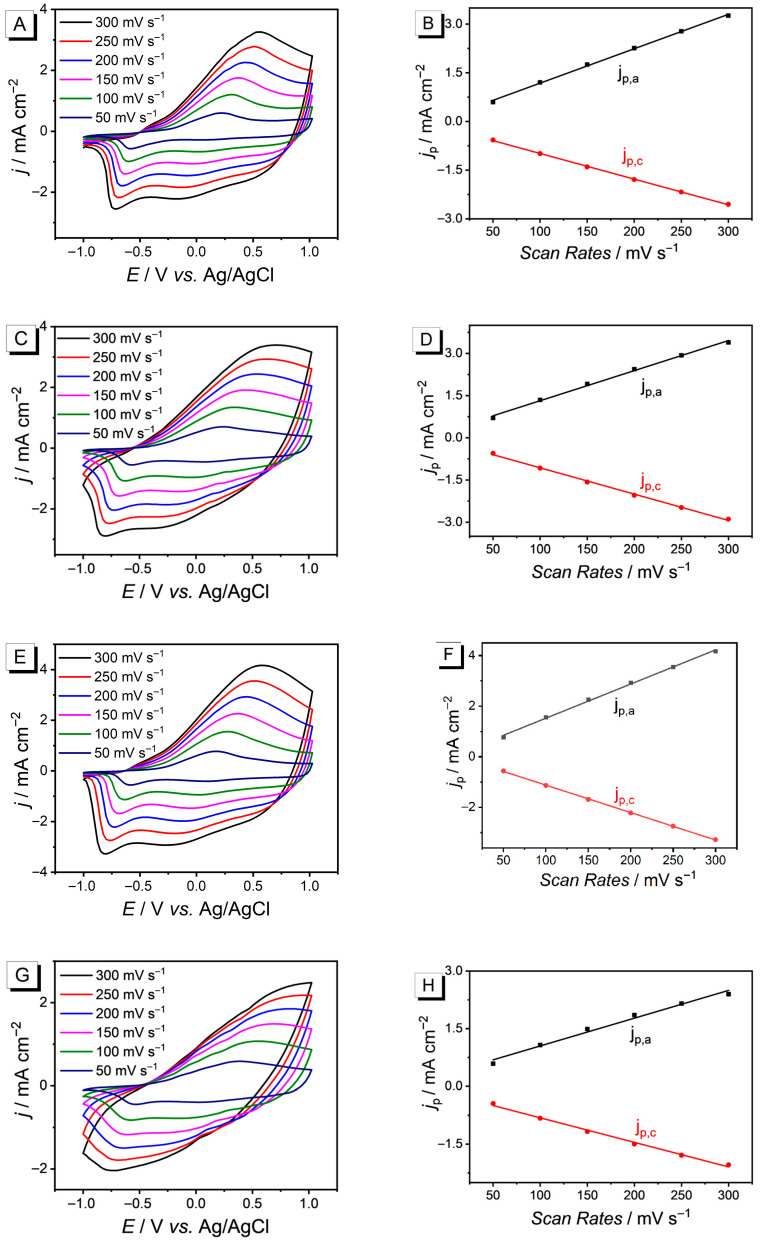
The cyclic voltammetric curves of PEDOT at room temperature in 0.1 M TBAPF_6_-CH_3_CN electrolyte (without monomer) at scan rates ranging from 50 to 300 mV·s^−1^ (left). The linear fitting of peak current density (*j*_p_) versus scan rate (right), where *j*_p,a_ and *j*_p,c_ represent the oxidation peak and reduction peak current densities, respectively: (**A**,**B**) PEDOT without post-treatment; (**C**,**D**) PEDOT treated with CB; (**E**,**F**) PEDOT treated with THF; (**G**,**H**) PEDOT treated with DMF.

In order to systematically investigate the redox activity and electrochemical stability of conductive polymer after annealing in solvents of varying polarity, this study employs cyclic voltammetry to compare the electrochemical behavior of PEDOT after annealing in CB, THF, and DMF in a monomer-free TBAPF_6_-CH_3_CN (0.10 mol·L^−1^) electrolyte system, as shown in Figure 5. Figure 5A,C,E,G show the cyclic voltammetric curves (CVs) of PEDOT at room temperature without post treatment as well as after annealing in CB, THF, and DMF at different scanning rates (from 300 mV·s^−1^ to 50 mV·s^−1^) in potential windows of −1 V to 1 V. Figure 5B,D,F,H are the linear fitting of peak current density (*j*_p_) versus scan rate (right).

All the polymers show distinct redox peaks with the similar CVs peak-shaped structure. The neutral chains of PEDOT (neutral state) lose electrons to form cation radicals (polarons) and bipolarons, accompanied by the migration of anions (PF_6_^−^) from the electrolyte into the film to balance the charge. This enhances the conductivity of the polymer in its oxidized state, resulting in an oxidation peak. The oxidized PEDOT acquires electrons, and the bipolarons/polarons are reduced back to the neutral chains. The anions migrate out of the film, resulting in a reduction peak, and PEDOT returns to its neutral state. The first cycle CVs of the polymer show that the redox peak potentials are at 0.2 V and −0.7 V. As the scan rate increases, the peak current density gradually increases, with the oxidation peak potential increasing and the reduction peak potential decreasing. All PEDOT polymers undergo potential shift during the process of peak potential increase. This phenomenon occurs mainly because as the scan rate increases, the system becomes less balanced and requires an additional overpotential to drive the reaction [30]. That is, the charge transfer process cannot keep up with the voltage change rate, which is a kinetic limitation causing peak potential migration [31]. However, the peak shape of PEDOT treated with DMF shows more significant changes, with an inconspicuous oxidation peak and a severe potential shift (Figure 5G). The highly crystalline and tightly π-π stacked PEDOT film induced by DMF elongates the diffusion path of PF_6_^−^. In order to charge fully within the specified time, the system has to increase the interfacial overpotential, which is manifested as the positive shift in the oxidation peak and the broadening of the peak shape. Particularly noteworthy is that the peak current density shows a good linear correlation (R^2^ ≈ 1) with the scan rate, indicating that the reaction is surface-controlled. Obviously, linear correlation of PEDOT treated with DMF (Figure 5H) is low-level than other three polymers. The differences in peak potentials after annealing with different solvents confirm that the volatility and polarity of the annealing solvents have a significant regulatory effect on the electrochemical behavior.

**Figure 6:**
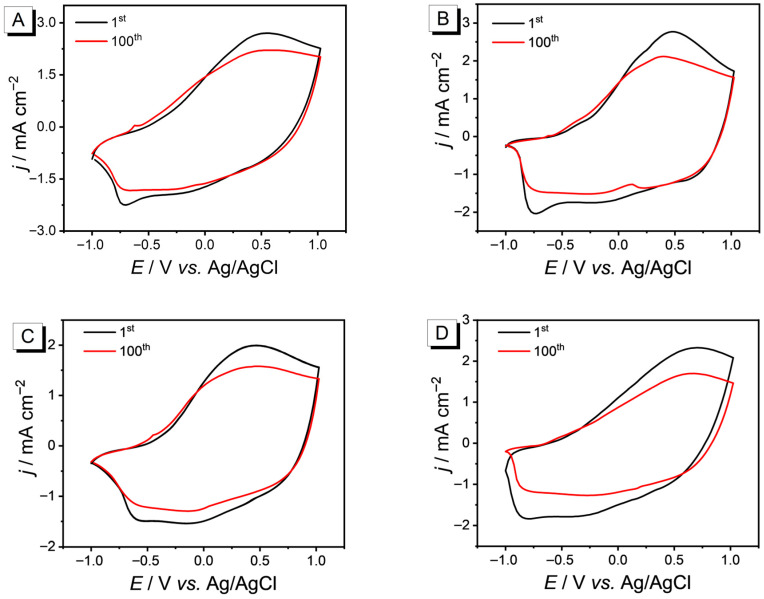
The electrochemical stability of four polymer films in a 0.1 M TBAPF_6_-CH_3_CN solution, with a scan rate of 200 mV·s^−1^: (**A**) PEDOT film without post-treatment; (**B**) PEDOT film treated with CB; (**C**) PEDOT film treated with THF; (**D**) PEDOT film treated with DMF.

In order to evaluate the electrochemical stability of PEDOT, cyclic voltammetry at a scan rate of 200 mV·s^−1^ was employed to study long-term testing in a solution of TBAPF_6_-CH_3_CN (0.10 mol·L^−1^) electrolyte only. As shown in Figure 6, after 100 cycles, the film retained 92.8% of its original electrical activity at room temperature. Following CB and THF annealing, it retained 86.28% and 86.06%. The annealing process promoted the rearrangement of PEDOT chains, forming a more ordered and denser film. This enhances crystallinity and conductivity but reduces ionic permeability. After DMF annealing, 74.07% of the original electrical activity was retained. The strong crystallinity may have affected the PF_6_^−^ doping of the PEDOT film [32].

### 3.5. Electrochromic Performance

#### 3.5.1. Spectroelectrochemistry

**Figure 7:**
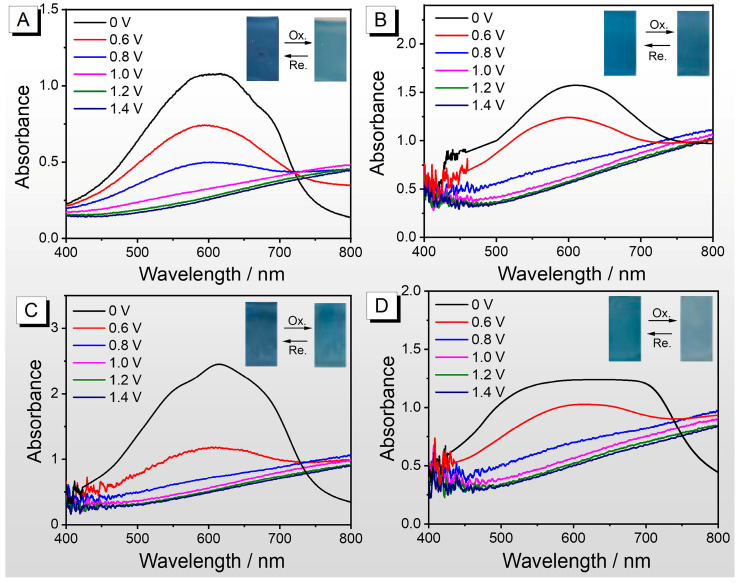
Spectral electrochemistry characterization of four polymer films in a 0.1 M TBAPF_6_-CH_3_CN solution (without monomer): (**A**) PEDOT film without post-treatment; (**B**) PEDOT film treated with CB; (**C**) PEDOT film treated with THF; (**D**) PEDOT film treated with DMF.

As shown in Figure 7, the spectroelectrochemical profiles of all polymers display a similar contour, accompanied by a characteristic absorption peak at around 600 nm and an isosbestic point at around 730 nm. PEDOT without post-treatment (Figure 7A) and treated with THF (Figure 7C) show more similar spectral changes versus voltage and closer film color variations, PEDOT treated with CB (Figure 7B) and treated with DMF (Figure 7D), due to the cracked film morphology and higher crystallinity (Figure 4), exhibit differences in spectra and color changes. Film cracking disrupts its continuity and conductive network, impeding charge transport, while high crystallinity leads to increased chain rigidity and reduced free volume, both of which hinder ion insertion and extraction during the doping/undoping process [32]. Taking Figure 7A as an example, let us interpret the variation in PEDOT’s spectrum with voltage. As a cathodic coloration material, PEDOT exhibits a deep blue color in its reduced state. As the voltage increases, the absorption intensity of the characteristic absorption peak at 600 nm decreases, and new absorption peaks appear after 800 nm due to the generation of polarons. When the voltage is sufficiently high, the PEDOT film turns light blue with significantly enhanced transmittance, and the 600 nm absorption peak disappears, indicating that PEDOT has completed the oxidation process [33]. Upon reducing the voltage, the film changes from light blue to deep blue, completing the reduction process.

#### 3.5.2. Dynamics

**Figure 8:**
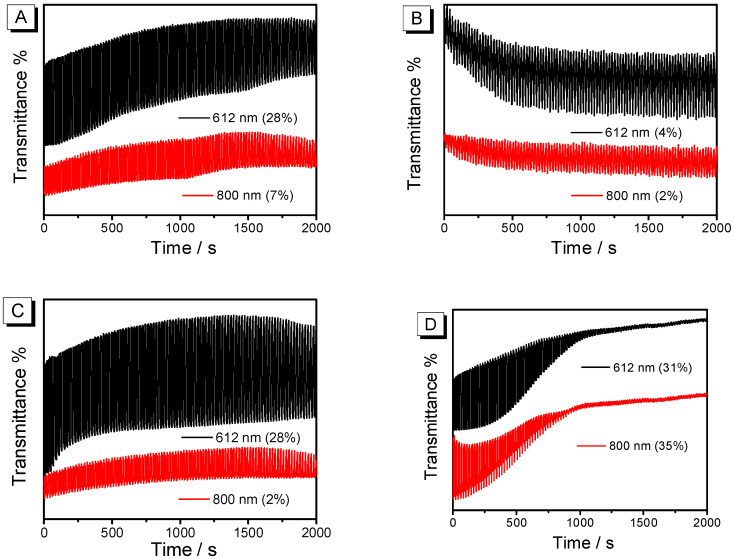
Time–transmittance curves of four polymer films tested in 0.1 M TBAPF_6_-CH_3_CN solution (without monomers): (**A**) PEDOT films without post-treatment; (**B**) PEDOT films treated with CB; (**C**) PEDOT films treated with THF; (**D**) PEDOT films treated with DMF.

**Figure 9:**
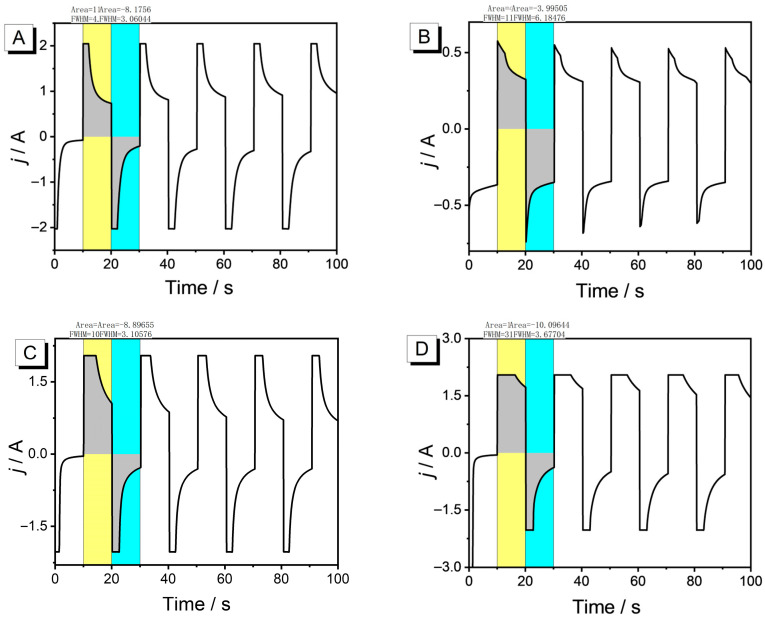
Corresponding chronoamperometry of (**A**) PEDOT films without post-treatment; (**B**) PEDOT films treated with CB; (**C**) PEDOT films treated with THF; (**D**) PEDOT films treated with DMF.

To objectively evaluate the electrochromic properties of the material, this study utilizes the second-order chronoamperometry method to detect the changes in optical transmittance (Figure 8) and electrochemical properties (Figure 9, injected/ejected charge as a function of electrode area) of the film in both doped (the gray area portion enveloped by the yellow region) and undoped states(the gray area portion enveloped by the sky blue region). We systematically summarized the key electrochromic parameters in Table 1, including optical contrast (*ΔT*), response time (*t*), and coloration efficiency (*CE*).

The PEDOT film without post-treatment (Figure 8A) and treated with THF (Figure 8C) exhibit similar *ΔT*, both reaching 28% at 612 nm, which is highly consistent with the performance of spectroelectrochemistry. In terms of kinetic stability, after 2000 s, the *ΔT* of the PEDOT film without post-treatment drops to 68.6%, while the PEDOT treated with THF shows higher stability, maintaining 82% of its initial state. It also achieves the highest *CE* of 184 cm^2^ C^−1^. This is mainly because, after THF annealing, its moderate volatility allows the PEDOT chains enough time to rearrange, forming a higher degree of π-π order, which provides continuous electronic pathways [34]. In addition, the trace amount of residual THF plasticizes the crystal boundaries, which can buffer the volume changes during doping/undoping and suppress cracking, jointly endowing the film with high optical contrast and high *CE*. During the annealing process, the large contraction rate of CB vapor leads to stress concentration within the film, easily forming micrometer-scale cracks (Figure 5B). These cracks disrupt the continuity of the film, creating “electrochemical dead zones,” which cannot be fully doped/undoped and do not contribute to light modulation, severely diluting the overall *ΔT* (Figure 8B). And due to the poor performance, we did not calculate the response time and *CE*. High-polarity solvents DMF lead to excessive crystallinity in the self-assembly of polymer films, resulting in dense, rigid, and defect-ridden aggregate structures. The restricted movement of polymer chains in highly crystalline regions narrows and twists ion channels, reducing the reversibility of doping/undoping [32], thereby lowering *CE* and compromising stability (Figure 8D). All PEDOTs exhibited fast response time within 2 s; meanwhile, their oxidation time is faster than the reduction time. From the perspective of kinetic test performance, solvent-assisted molecular self-assembly plays a crucial role in optimizing the electrochromic behavior of PEDOT. Solvent annealing effectively modulates the aggregate structure of polymer chains, affecting the crystallinity, pore size distribution, and interfacial contact of the film [35], thereby altering ion diffusion pathways and electrochromic kinetics during electrochemical processes.

## 4. Conclusions

In summary, solvent annealing plays a crucial role in the properties of electrochromic polymers. This study focuses on investigating the effects of organic solvents with different polarities on the performance of PEDOT. After CB and THF solvent annealing, PEDOT exhibits similar crystallinity, which is reflected in the relatively gentle crystallization peaks on the DSC curves. Based on this, both polymers show similar electrochemical stability. However, during the annealing process, the large contraction rate of CB vapor leads to stress concentration within the film, easily forming micrometer-scale cracks. These cracks disrupt the continuity of the film, creating “electrochemical dead zones,” which cannot be fully doped/undoped and do not contribute to light modulation, severely diluting the overall optical contrast, response time, and coloration efficiency. PEDOT annealed with DMF shows the highest crystallinity, with a sharp melting peak on the DSC curve and visible crystallites in morphology. This highly crystalline structure is not suitable for the doping and undoping processes of PF_6_^−^ ions, resulting in poor electrochemical and electrochromic kinetic stability. Different polarities and volatilities of solvents induce the self-assembly behavior of PEDOT, producing PEDOT films with different aggregate states, which makes the electrochemical and electrochromic properties of PEDOT vary greatly.

## Figures and Tables

**Table 1:** Electrochromic parameters of PEDOT.

PEDOT	Wavelength	∆*T* (%)	Response Time (s)		*CE* (cm^2^ C^−1^)	
	(nm)		Oxidation	Reduction	Oxidation	Reduction
Without post-treatment	612 nm	28%	1.1 s	1.4 s	64	93
Treated with CB	612 nm	4%	--	--	--	--
Treated with THF	612 nm	28%	1.1 s	2 s	113	184
Treated with DMF	612 nm	31%	1.2 s	1.9 s	69	135

## Data Availability

Data will be made available on request.
